# Embolic Shotgun Pellet to the Left Middle Cerebral Artery Causing Hemiplegia and Aphasia With Near Complete Clinical Recovery on Nonoperative Management

**DOI:** 10.7759/cureus.11677

**Published:** 2020-11-24

**Authors:** Mark Mingo, Devin Cao, Chizoba Ezepue, Reyanna Massaquoi, Mehrdad Sehi

**Affiliations:** 1 Radiology, St. Louis University Hospital, St. Louis, USA; 2 Neurology, St. Louis University Hospital, St. Louis, USA; 3 Radiology/Neuroradiology, St. Louis University Hospital, St. Louis, USA

**Keywords:** gunshot wound, bullet emboli, middle cerebral artery, shotgun, embolic, transient hemiplegia

## Abstract

A 28-year-old male presented after gunshot injury to his right side from a shotgun. He had no prior history of gunshot injury and no neurologic deficits on presentation. Initially, non-contrast computed tomography (CT) scans of the head, face, chest, abdomen, and pelvis demonstrated multiple pellets lodged in the patient’s right upper extremity, face, abdomen, and right hemithorax which penetrated the right lung. A shotgun pellet was also found in the region of the left middle cerebral artery (MCA) on the head CT without contrast with no skull fracture or intracerebral hemorrhage. The patient subsequently developed right hemiplegia and expressive aphasia approximately 48 hours after the trauma. CT angiography (CTA) of the head and neck with perfusion at that time demonstrated ischemic penumbra and the location of the pellet to be in the distal left M1 branch. No intervention was performed given the location. The patient clinically improved without intervention. This is an uncommon injury and outcome for embolization of a foreign body.

## Introduction

Missile embolization to the cerebral circulation remains an unusual and rare complication of gunshot wounds that usually has devastating outcomes [1-4]. Embolism from shotgun wounds are attributed to the combination of small pellet size, wide surface area of injury, and low velocity projectiles whose low kinetic injury allows for incomplete perforation of the affected vessel, thus trapping it in systemic circulation [4-6]. The current management guidelines of pellet embolization to the cerebral vasculature remain limited with the literature reporting surgery, anticoagulation, and observation, all as acceptable treatment options [1-9]. We present a case of an embolic shotgun pellet to the left middle cerebral artery (MCA) causing transient neurologic symptoms which was managed with anti-platelet therapy and observation. The exact route of the embolic pellet was unable to be confirmed definitively, but a few possibilities are entertained after workup excluded some of the initial theories.  

## Case presentation

**Figure 1:**
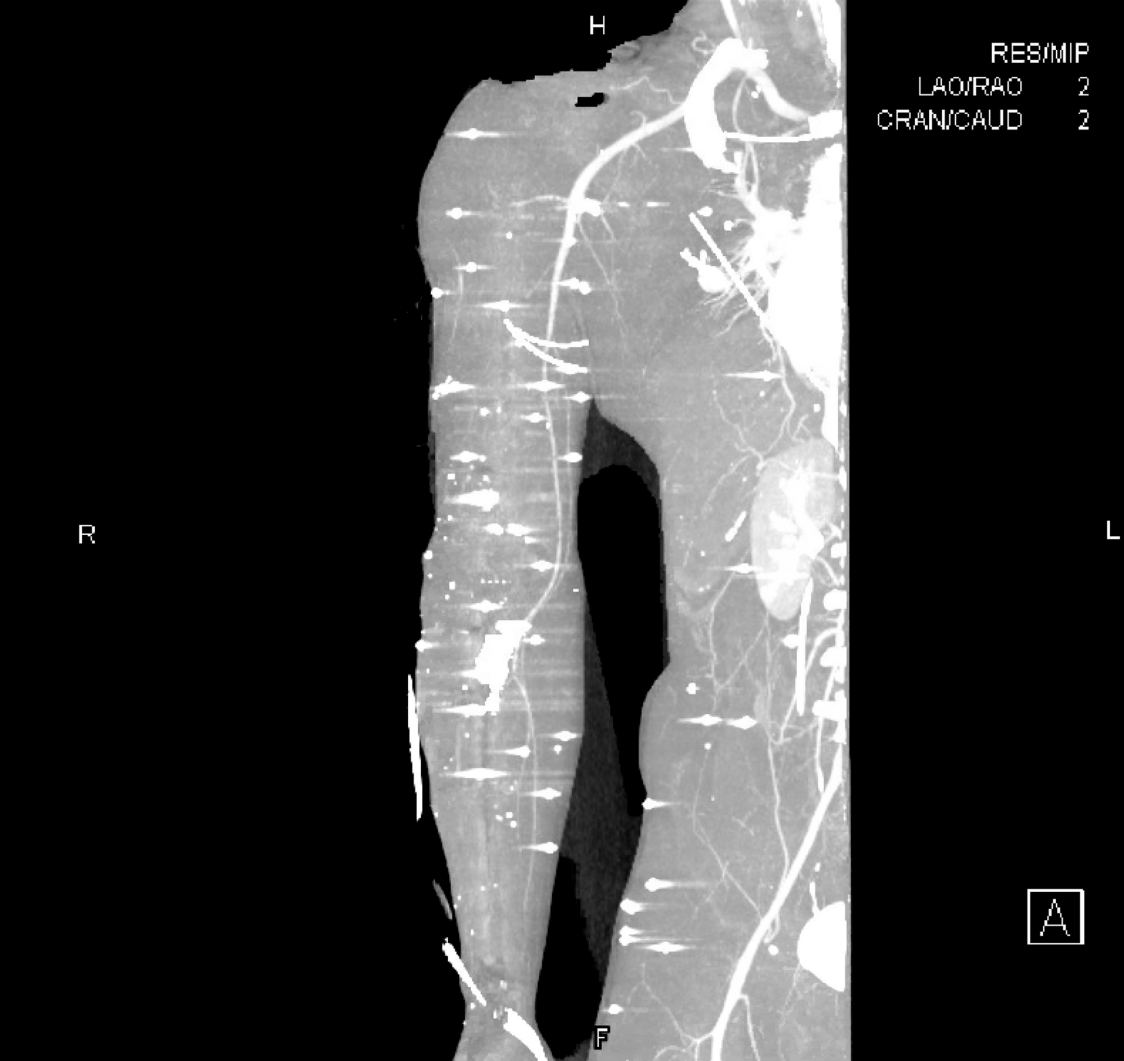
CTA MIP right upper extremity Image shows pellets with streak artifacts of the right upper extremity and right abdomen

**Figure 2:**
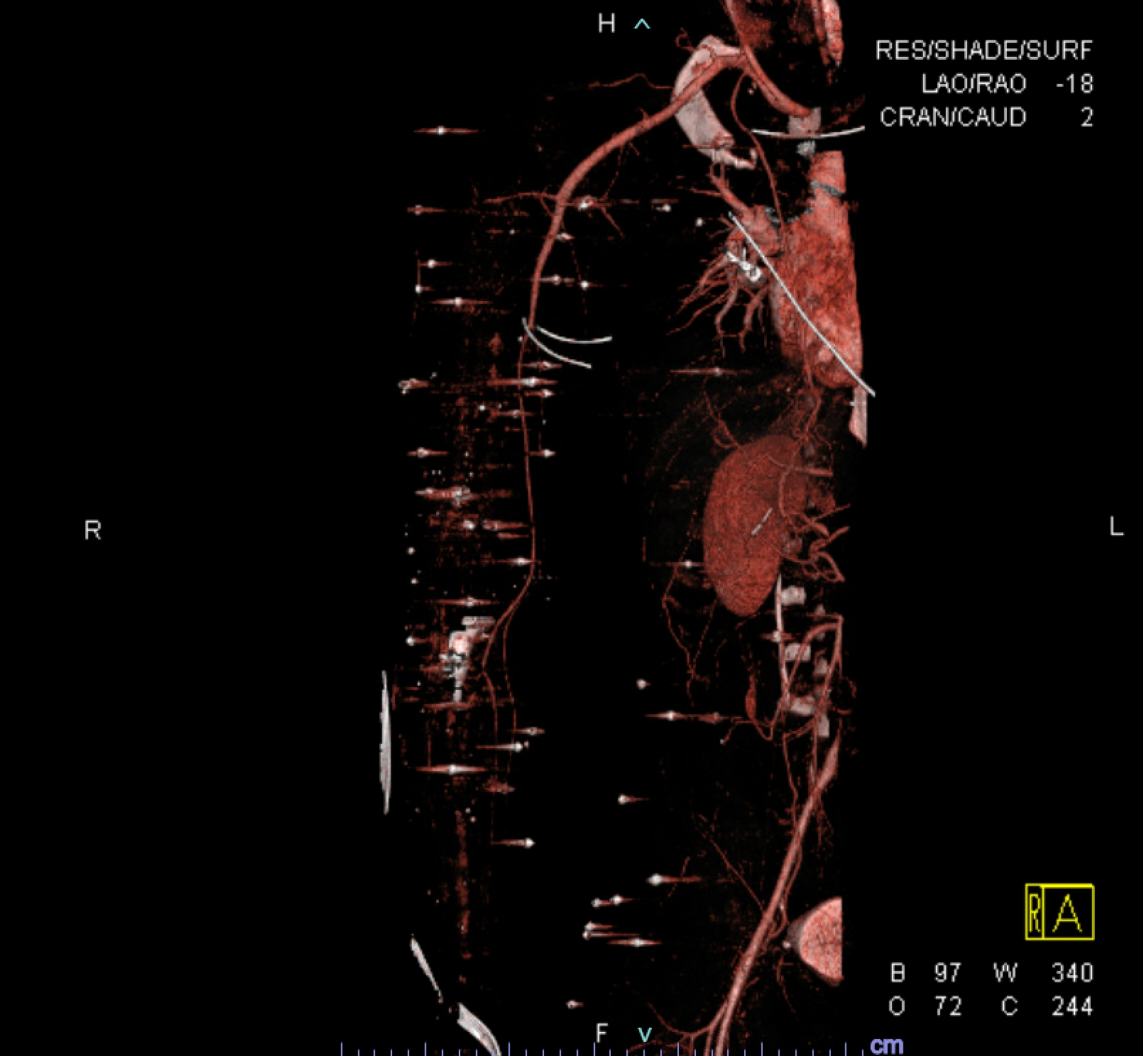
CTA 3D reformat right upper extremity Image shows pellets with streak artifacts of the right upper extremity and right abdomen

**Figure 3:**
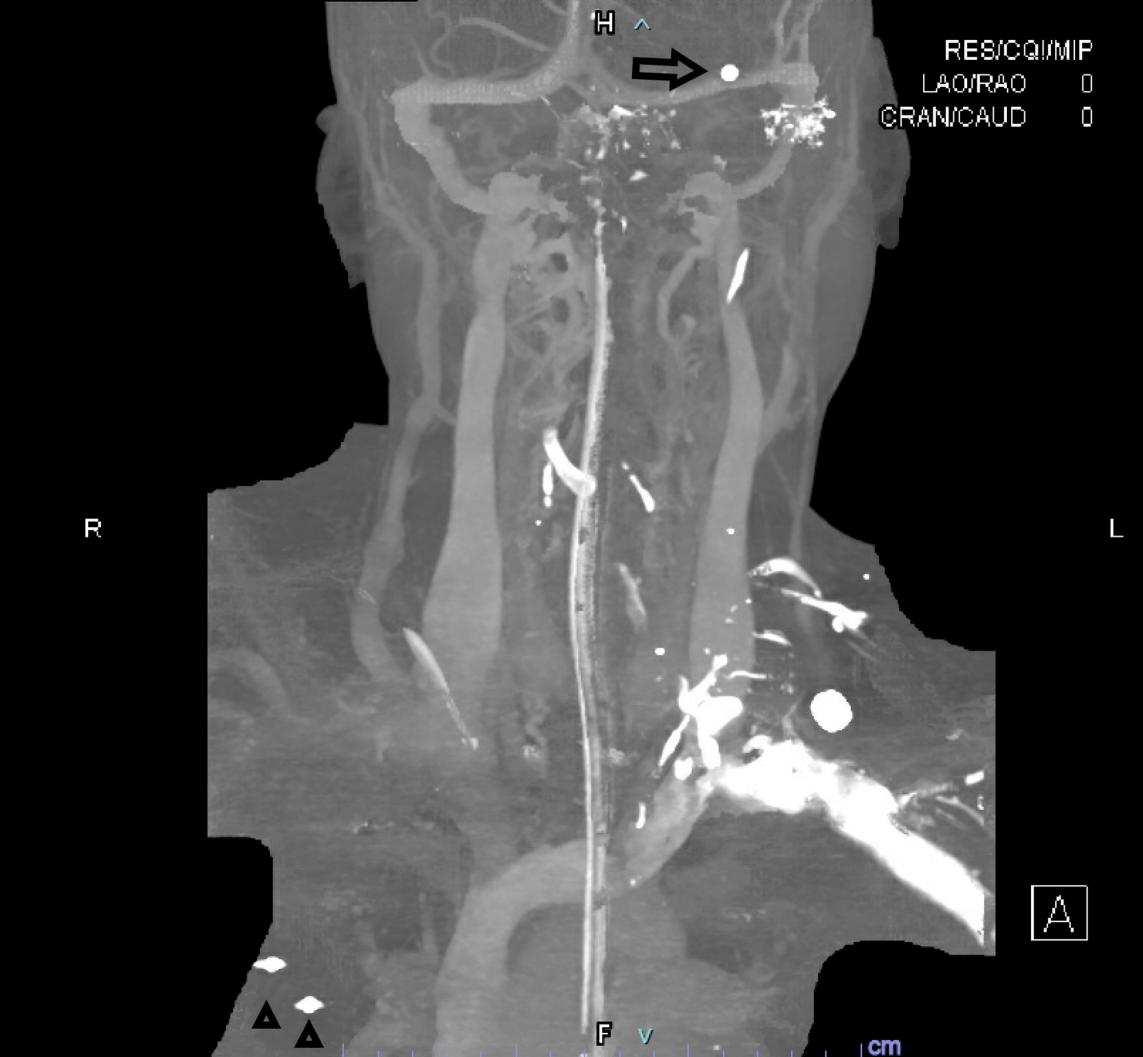
CTA head and neck coronal MIP Arrow=Pellet in location of left MCA Arrowheads=Pellets in the right upper lobe

**Figure 4:**
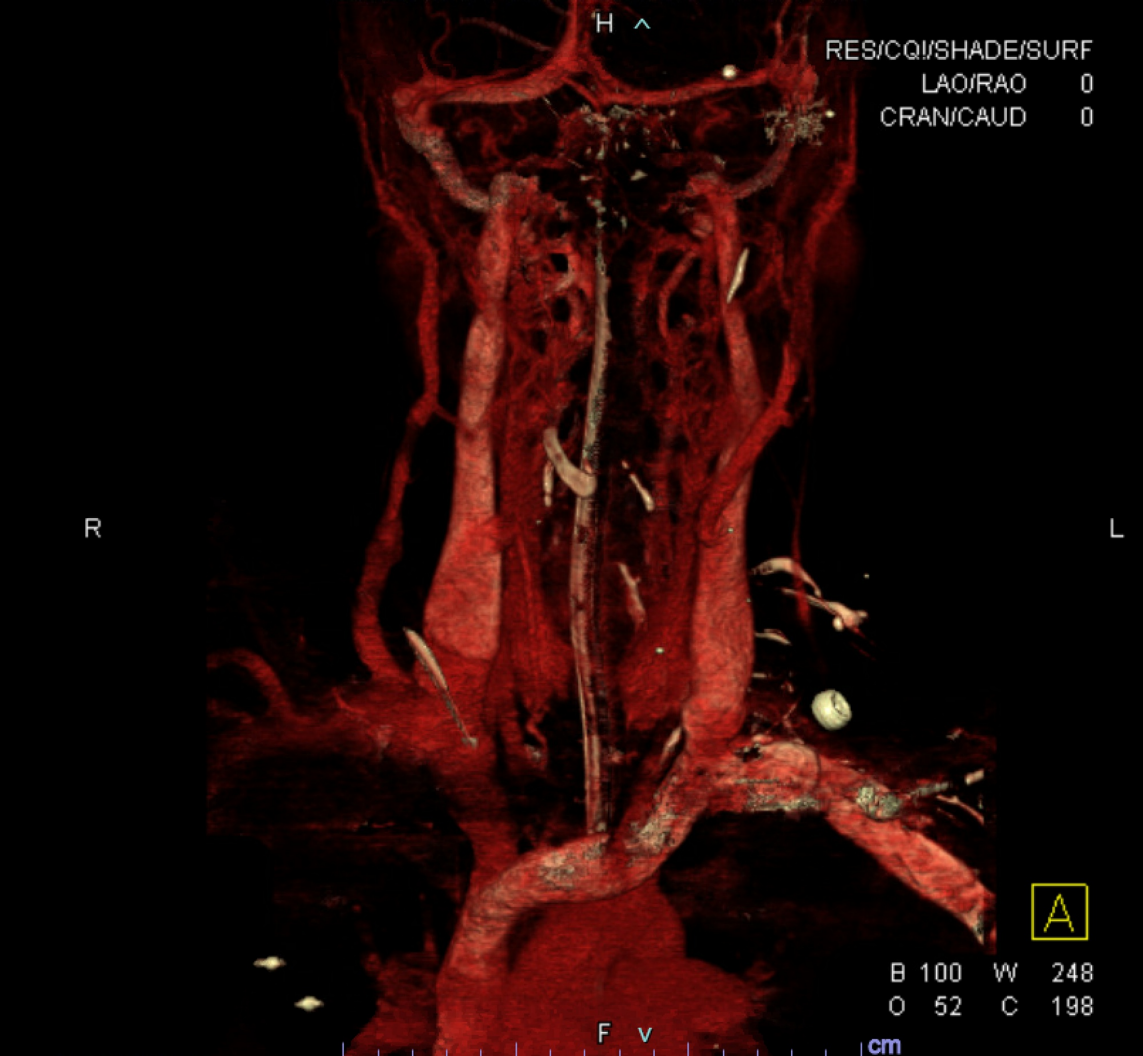
CTA head and neck coronal 3D reformats

An otherwise healthy 28-year-old male was brought to the emergency department of Saint Louis University Hospital after being shot through a glass window with a shotgun. On arrival, he was conscious with multiple retained buckshot pellets in the right face, right upper extremity, right chest and abdomen (figures 1-4). The initial neurologic examination was normal with no focal deficit.    

**Figure 5:**
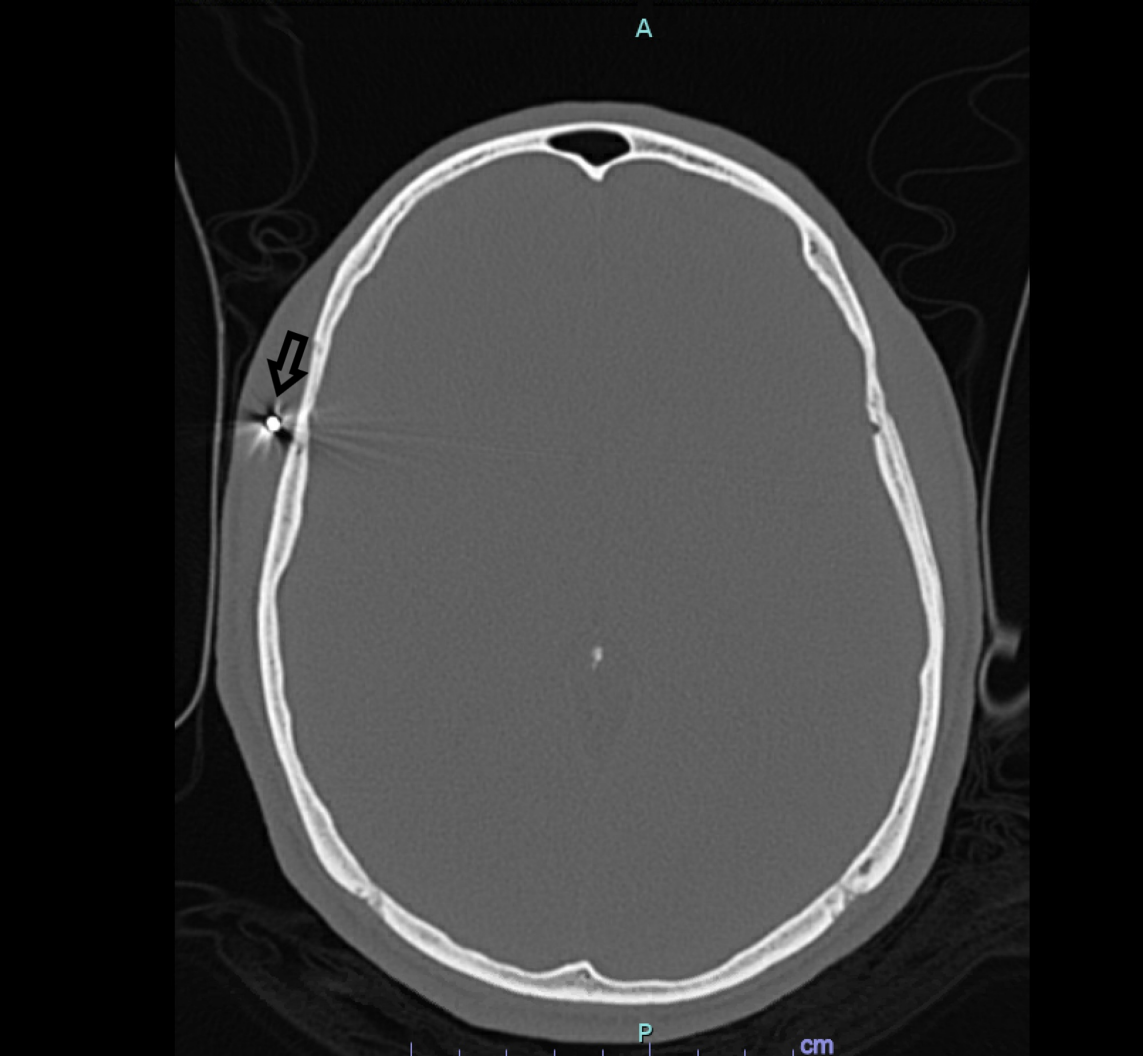
CT head without contrast Arrow=Pellet in right facial soft tissues

**Figure 6:**
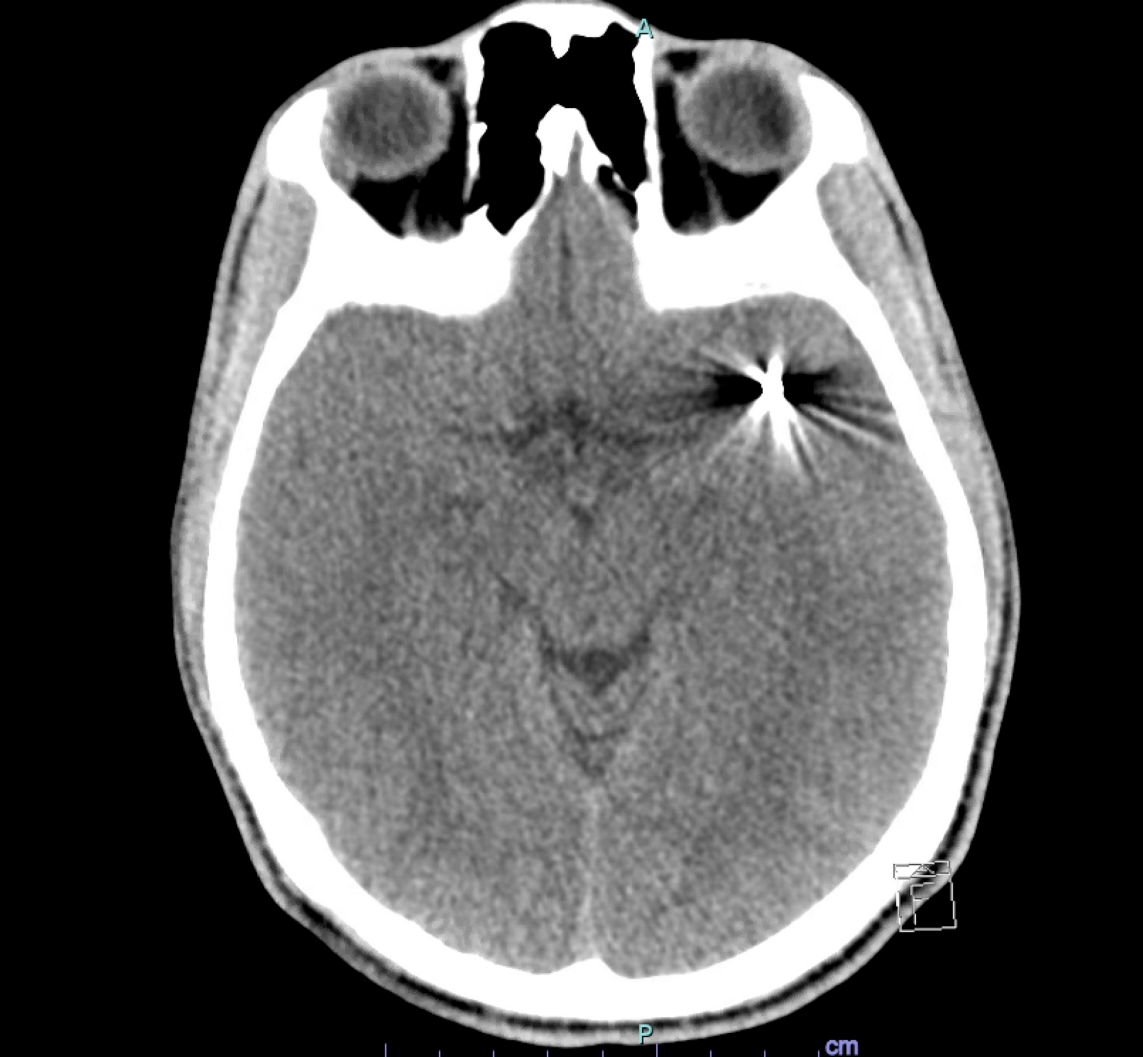
CT head without contrast demonstrating a pellet in the expected location of the left MCA

Initial CT brain without contrast showed multiple shotgun pellets; these were located along the left temporal lobe in the Sylvian fissure, in the right frontal scalp, and in the soft tissues of the face without any evidence of skull fracture or intracranial hemorrhage (figure 5-6). The route of the intracranial pellet was unknown at the time and was thought to possibly be from prior gunshot since there were no large arterial injuries identified and the patient was neurologically intact. No visible entry point via the skull was identified. No other intracranial abnormalities were identified. 

The patient underwent an emergent exploratory laparotomy for primary repair of penetrating bowel injuries from the ballistics and cholecystectomy.   

**Figure 7:**
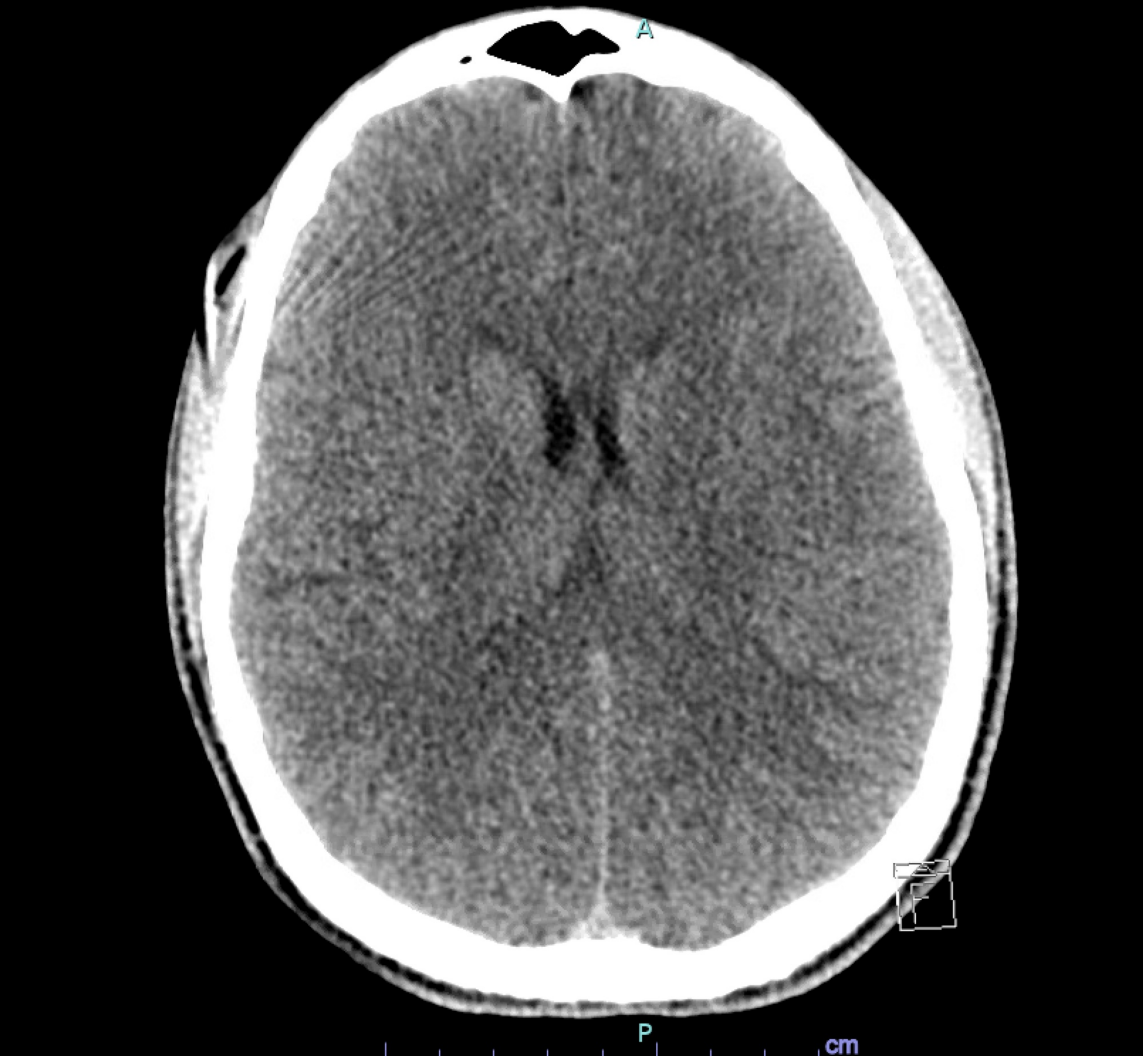
Normal appearing parenchyma at the level of the lateral ventricles

**Figure 8:**
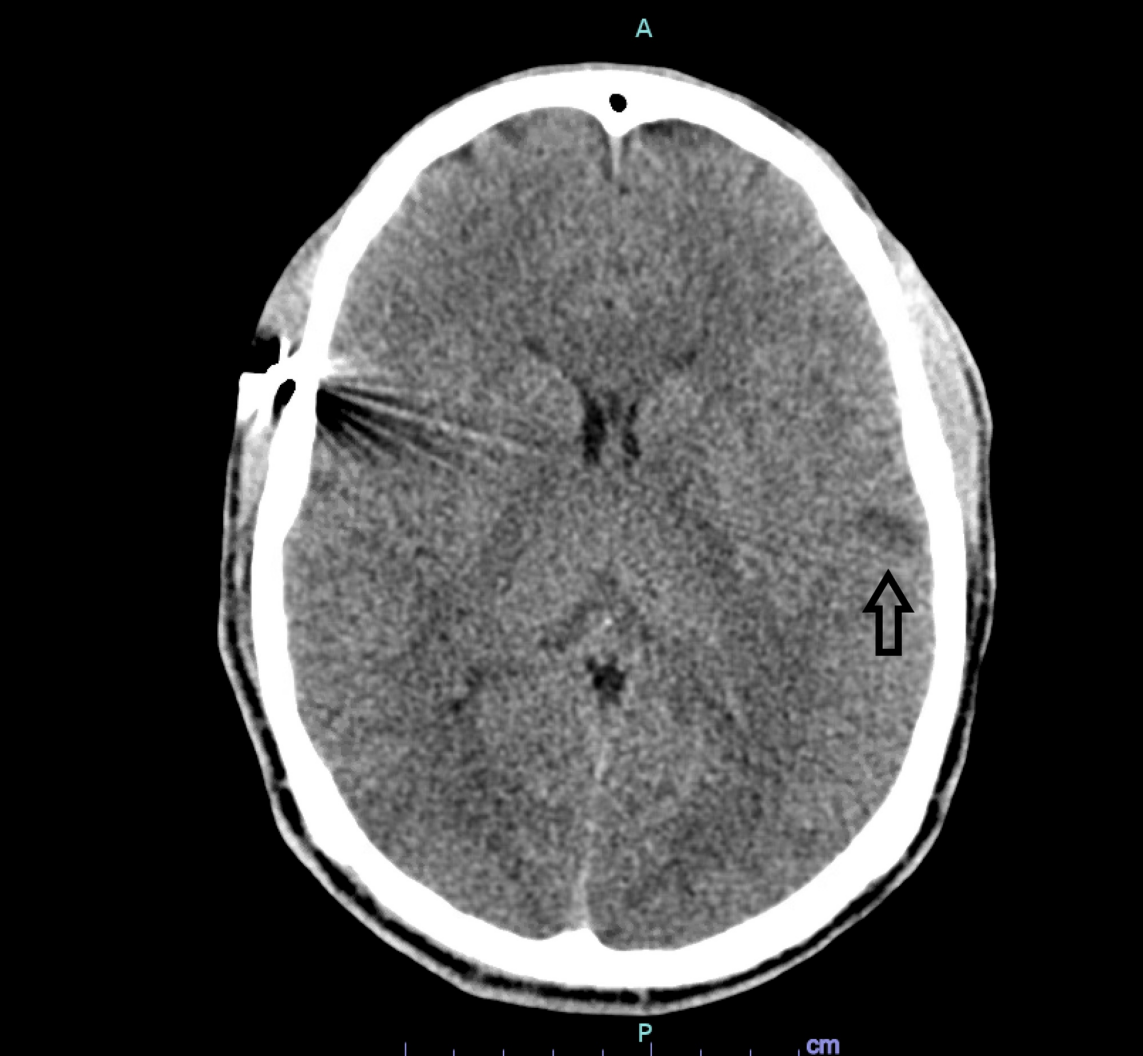
CT head with new area of hypodensity in the left frontal lobe

**Figure 9:**
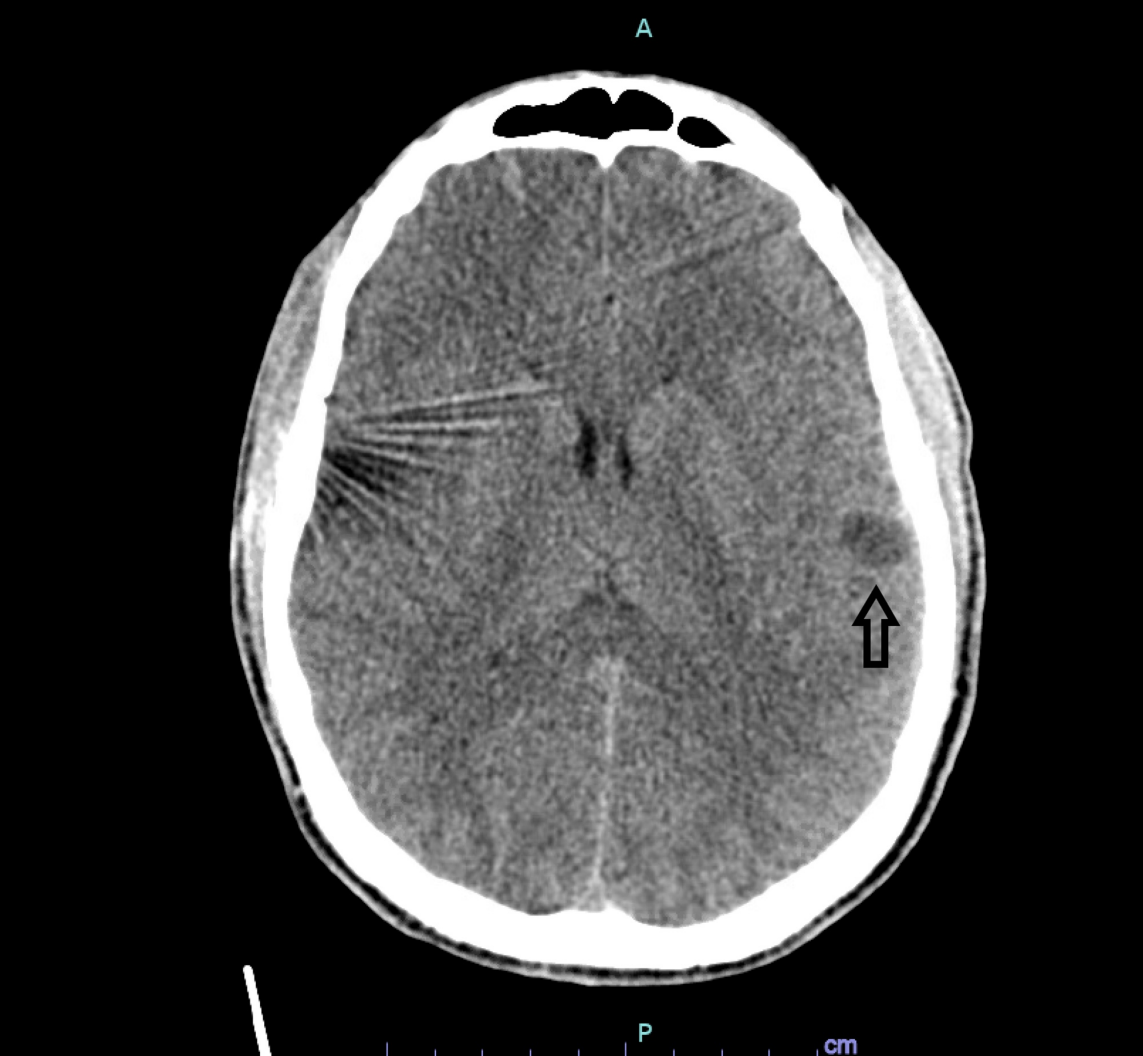
CT head demonstrating increased hypodensity in the left frontal lobe

The patient remained intubated and sedated postoperatively. After extubating the patient, approximately 48 hours after admission, his neurologic examination was significant for new onset right hemiplegia and expressive aphasia. Repeat CT head revealed new area of hypodensity in the left frontal lobe, which persisted on repeat CT head the following day (figures 7-9) . CT angiography of the head with perfusion and CTA of the neck were ordered emergently.

**Figure 10:**
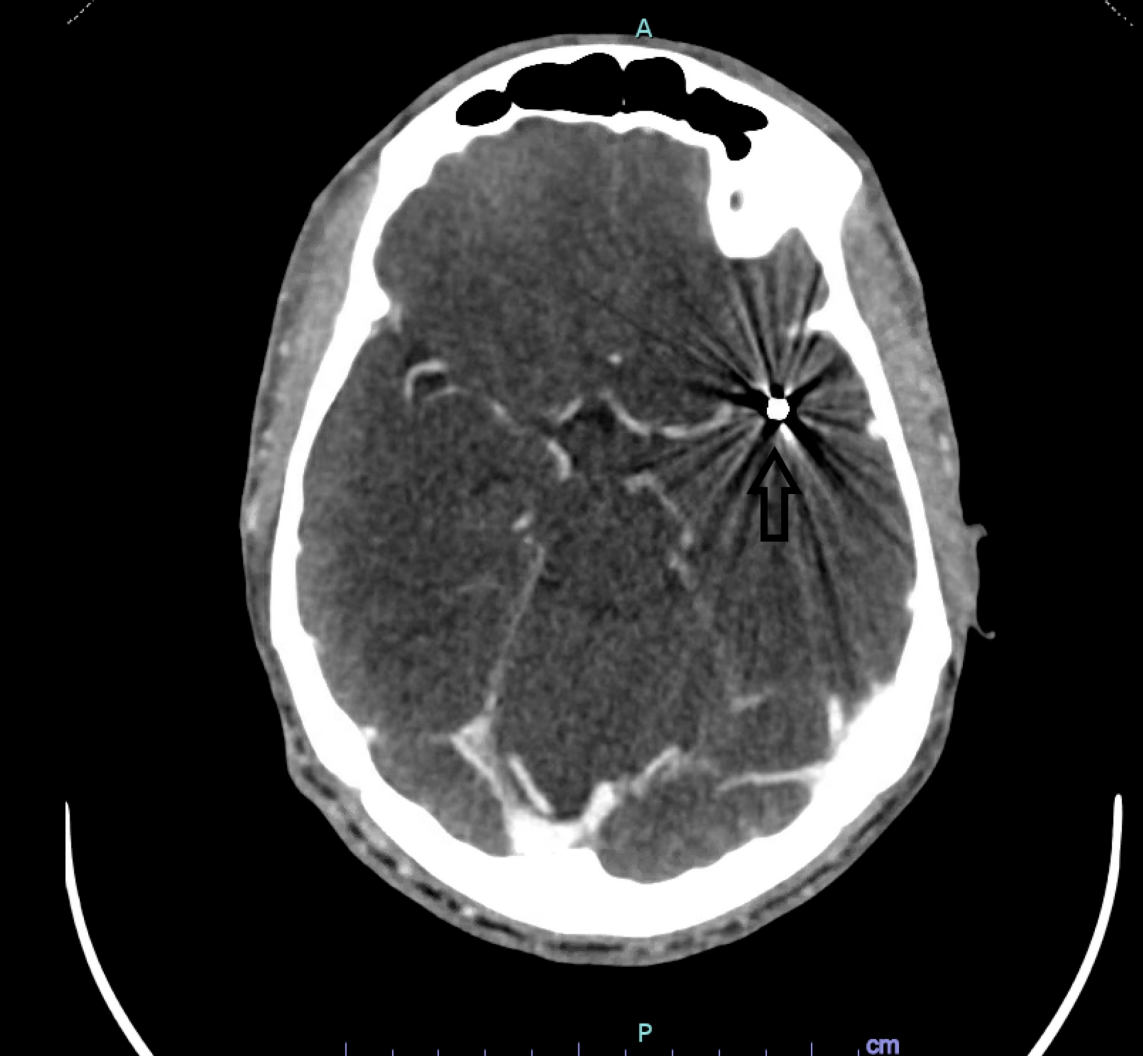
CTA head demonstrating pellet in left MCA distal M1 or proximal M2 branch (streak artifact limits exact localization)

**Figure 11:**
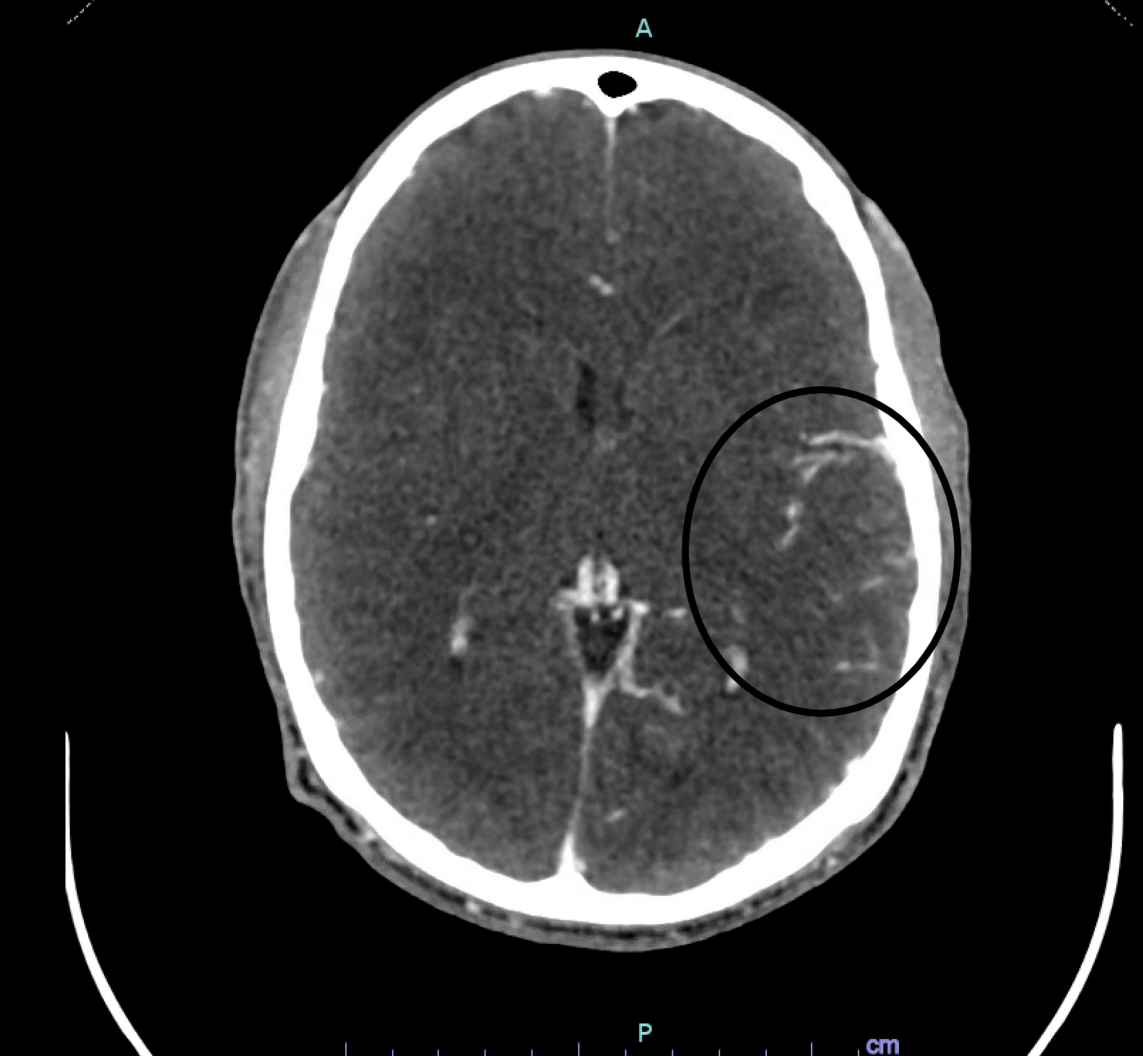
CTA head demonstrating dilation of the distal branches of the left MCA territory

CT angiography of the head and neck revealed a shotgun pellet at the bifurcation of the left MCA with dilation of the MCA branches distal to the pellet (figures 10-11). This was thought to likely be compensatory from partial occlusion caused by the pellet. Again, no visible entrance of the bullet through the skull or cervical arteries were identified.  

**Figure 12:**
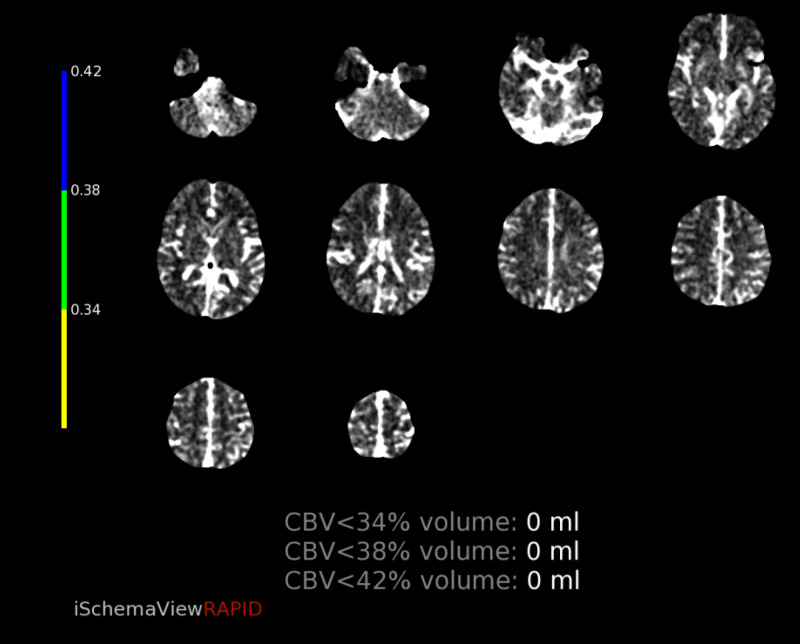
CT perfusion demonstrating normal CBV CBV=Cerebral blood volume

**Figure 13:**
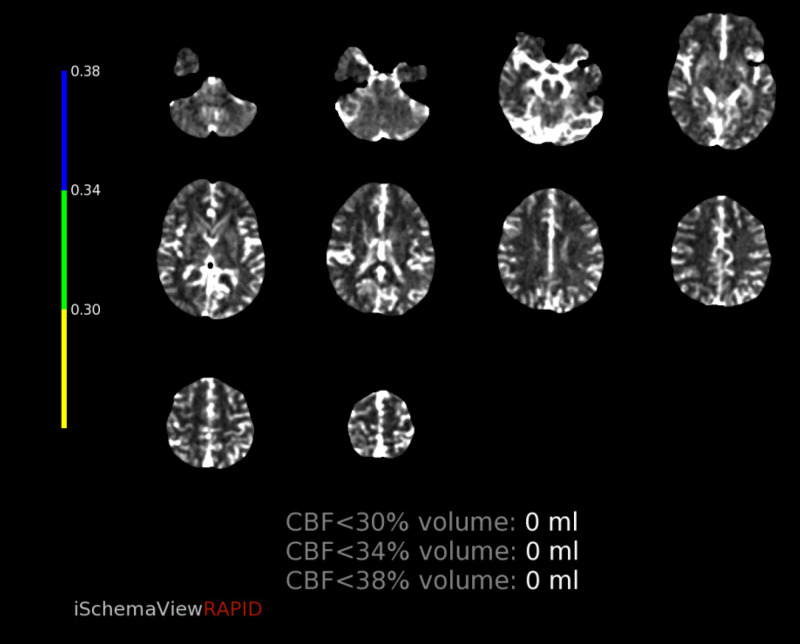
CT perfusion demonstrating normal CBF CBF=Cerebral blood flow

**Figure 14:**
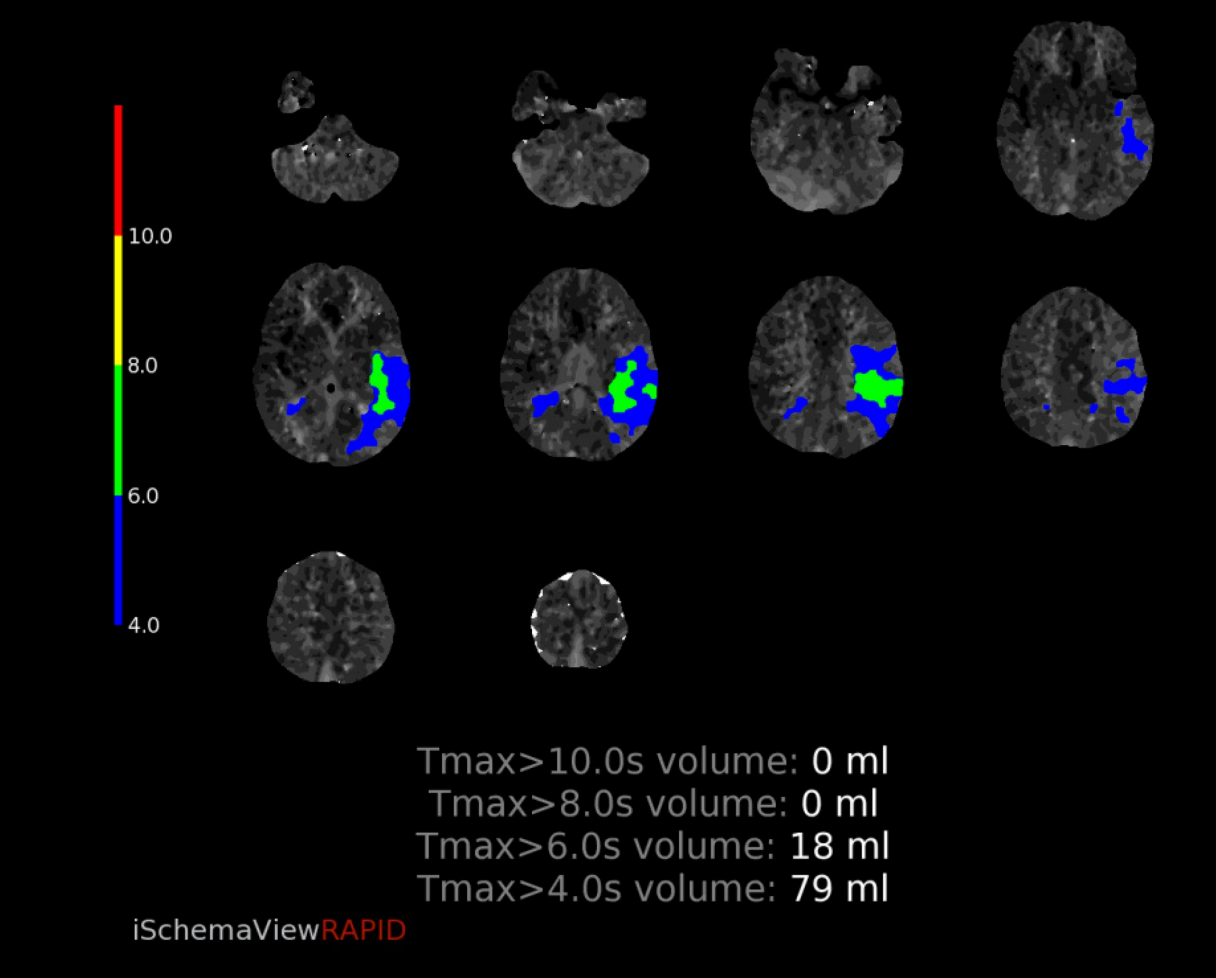
CT perfusion demonstrating elevated Tmax in the left frontal lobe Tmax=Time-to-Maximum

**Figure 15:**
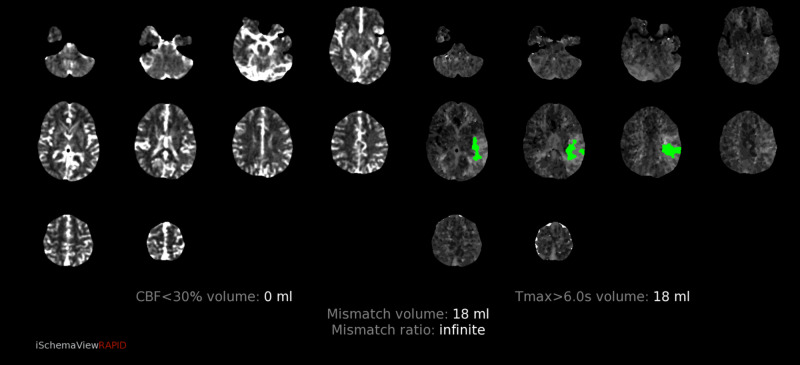
CT perfusion demonstrating mismatch volume of 18mL

**Figure 16:**
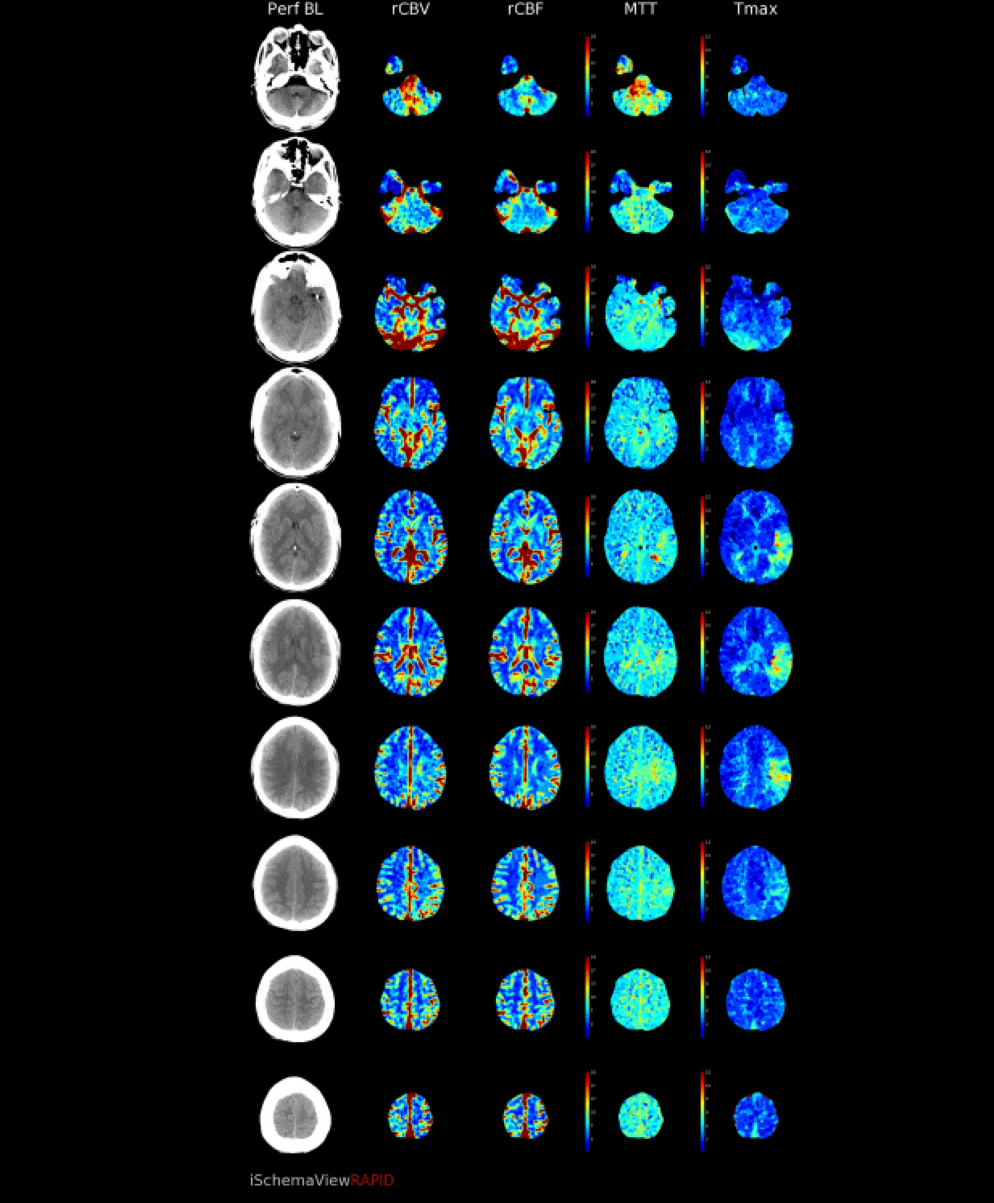
CT perfusion demonstrating preserved rCBV, rCBF, and MTT with elevated Tmax in the left MCA territory of the left frontal lobe

CT perfusion revealed abnormalities in a region supplied by the left MCA. The cerebral blood volume (CBV) and cerebral blood flow (CBF) were normal, however there was elevated Time-to-Maximum (Tmax) which was thought to correlate with compensatory dilation of the distal MCA branches (figure 12-16).  Intervention was not pursued due to the patient’s labile hemodynamic status from other injuries and risk of complication. A transthoracic echocardiogram was obtained to look for a patent foramen ovale or atrial septal defect to exclude the possibility of a right-to-left shunt bypassing the lungs. The echocardiogram showed no abnormality. 

The patient was started on aspirin, and by the time of discharge to a rehabilitation center, the patient’s neurologic exam improved with minimal residual neurologic deficits. On his most recent neurology clinic visit, four months after hospital discharge, the patient’s only reported neurologic symptoms were chronic intermittent pain in the right upper and lower extremities. On objective physical examination, he was reported to have 6/8 strength of the right lower extremity. 

## Discussion

The lack of a definitive route of entry and embolization in the case of this patient prompted a brief review of the literature surrounding the mechanism of cerebral arterial embolization following gunshot wounds. In Kuo et.al, an analysis of 261 cases of civilian intravascular ballistic embolization revealed that a disproportionate number of emboli (69%) were associated with truncal entry wounds with roughly equal involvement of the left and right circulation. However, in cases where a centrally located embolus did migrate cranially, there was a greater propensity for right (74%) over left (26%) laterality [10]. Reported pathways commonly involve direct entry via the thoracic aorta, pulmonary veins, or left ventricle with less common etiologies including transseptal migration into the left ventricle or, even rarer, retrograde embolization. Additional factors to consider when assessing for possible routes of entry also include the firing distance, trajectory, velocity, and physical properties of the projectile itself [11,12]. 

In our patient, one hypothesized route of embolization is arterial injury of the pulmonary vasculature which drained into the left atrium, subsequently leading to the entry of the pellet into the left internal carotid artery where it eventually traveled to, and partially occluded, the left MCA at the distal M1 segment. An alternate hypothesis is entry of the pellet through a wound in the chest into the aortic arch or left common carotid artery creating a valve that prevented extravasation of blood into the mediastinum or neck and then through usual physics of blood flow the pellet gets propelled to the left ICA and through the carotid terminus to the distal M1 where it is trapped prior to the bifurcation. Given the negative transthoracic echocardiogram, a transseptal migration is unlikely to have occurred in our case. 

The optimal management of intracranial arterial pellet embolization continues to remain controversial. Appropriate neuroradiologic workup of gunshot wound patients with new neurologic deficits should include CT head for assessment of injury type and severity as well as for prognostication. CTA with perfusion may also be considered for evaluation of vascular territory anatomy and involvement. In a review of 30 cases, 4(13.4%) asymptomatic and 26 (86.6%) with some neurologic deficit, underwent conservative management - suggesting that intracranial pellets can be well tolerated [4]. Indications for surgical intervention in the literature have classically included preventing thrombus propagation and complete infarct, vascular erosion, and mitigating risk of infection [7,8]. The primary risk associated with surgical management was ballistic body migration and distal embolization, as was observed in 9% of retrieval attempts [4]. Additional factors which argue against surgical management included a stable neurologic deficit, presence of collateral circulation on arteriographic imaging, and a complete infarct [7,13]. Our case highlights the consideration of these factors in respect to overall prognosis when determining management.

## Conclusions

In summary, embolized pellet fragments to intracranial circulation following gunshot related thoracic injury, while having been occasionally reported in the past, continues to remain limited in the literature. This case distinguishes itself from those previously reported by its combination of delayed onset of presentation, proposed routes of injury, and clinical resolution of neurologic deficits with non-operative management. In patients with focal neurologic deficits who are not surgical candidates, anticoagulation and supportive treatment should still be considered.

## References

[1] Cogbill Cogbill, Thomas H. MD; Sullivan, Humbert G (1995). Carotid artery pseudoaneurysm and pellet embolism to the middle cerebral artery following a shotgun wound of the neck. J Trauma.

[2] Alsofrom DJ, Marcus NH, Seigel RS, Talbot WA, Akl BF, Schiller WR, Sklar DP (1982). Shotgun pellet embolization from the chest to the middle cerebral arteries. J Trauma.

[3] Dadsetan Dadsetan, M.R. M.R., Jinkins Jinkins, J.R J.R Peripheral vascular gunshot bullet embolus migration to the cerebral circulation. Neuroradiology.

[4] Vaquero-Puerta C, San Norberto EM, Merino B, González-Fajardo JA, Taylor J (2012). Shotgun wound and pellet embolism to the intracranial carotid artery. J Vasc Surg.

[5] Vedelago J, Dick E, Thomas R (2014). Look away: arterial and venous intravascular embolisation following shotgun injury. J Trauma Manag Outcomes.

[6] Gipe BT, Acker B, Smith R (1981). Delayed cerebral embolization of a shotgun pellet with fatal consequences. J Trauma.

[7] Stein M, Mirvis SE, Wiles CE III (1995). Delayed embolization of a shotgun pellet from the chest to the middle cerebral artery. J Trauma.

[8] Monterey M, Kerr K, Dannenbaum M, Chen PR, Blackburn S (2019). Open Surgery for Extraction of an Embolized Pellet in the Middle Cerebral Artery From a Shotgun Injury. Oper Neurosurg.

[9] McCague A, Kelly S, Wong DT (2013). Shotgun pellet embolization to the middle cerebral artery. Am Surg.

[10] Kuo AH, Gregorat AE, Restrepo CS, Vinu-Nair S (2019). Systematic review of civilian intravascular ballistic embolism reports during the last 30 years. J Vasc Surg.

[11] Vakil MT, Singh AK (2017). A review of penetrating brain trauma: epidemiology, pathophysiology, imaging assessment, complications, and treatment. Emerg Radiol.

[12] Chao J, Barnard J, deJong JL, Prahlow JA (2018). A case series of anterograde and retrograde vascular projectile embolization. Acad Forensic Pathol.

[13] da Costa LB, Wallace MC, Montanera W (2006). Shotgun pellet embolization to the posterior cerebral circulation. AJNR Am J Neuroradiol.

